# No evidence for the association of DRD4 with ADHD in a Taiwanese population within-family study

**DOI:** 10.1186/1471-2350-6-31

**Published:** 2005-09-05

**Authors:** Keeley-Joanne Brookes, Xiaohui Xu, Chih-Ken Chen, Yu-Shu Huang, Yu-Yu Wu, Philip Asherson

**Affiliations:** 1 MRC Social Genetic Developmental Psychiatry Centre, Institute of Psychiatry, London UK; 2 Department of Psychiatry, Chang Gung Memorial Hospital, Taiwan

## Abstract

**Background:**

Attention Deficit Hyperactivity Disorder (ADHD) is a prevalent and highly heritable childhood disorder. The dopamine D4 receptor (DRD4) gene has shown a genetic association with ADHD in Caucasian populations with meta-analysis indicating a small but significant effect across datasets. It remains uncertain whether this association can be generalised to non-Caucasian ethnic groups. Here we investigate two markers within the DRD4 gene in a Taiwanese population, the exon 3 variable number tandem repeat (VNTR) and a 5' 120 base-pair duplication.

**Methods:**

Within-family transmission disequilibrium tests of association of the 5' 120 base-pair duplication, and exon 3 VNTR in a Taiwanese population.

**Results:**

No evidence of association of ADHD with either polymorphism in this population was observed.

**Conclusion:**

The DRD4 gene markers investigated were not found to be associated with ADHD in this Taiwanese sample. Further work in Taiwanese and other Asian populations will therefore be required to establish whether the reports of association of DRD4 genetic variants in Caucasian samples can be generalised to Asian populations.

## Background

Attention Deficit Hyperactivity Disorder (ADHD) is one of the most prevalent and heritable childhood behavioural disorders. Progress in identifying some of the genes involved in ADHD susceptibility has been relatively fruitful over the past decade by screening genetic variants that lie within or close to genes that regulate neurotransmitter systems, particularly dopamine pathway genes [1]. One of the more consistent findings is the association with the 7-repeat allele of a 48-base pair variable number tandem repeat (VNTR) in exon 3 of D4 receptor gene (DRD4) although there are a number of negative reports and discrepancies between case control and within family studies [2-22]. Meta-analyses of published and unpublished data indicate a small but significant association across datasets with no evidence of heterogeneity [23,24].

Other genetic variants in the DRD4 5'-regulatory region have also been reported to be associated with ADHD. Of particular interest is a 120-bp duplication that has been associated with ADHD in two studies [25,26] although negative studies have also been reported [27-29,20]. Recently our group investigated the functional significance of this repeat marker using in vitro reporter gene assays and found that the long allele conferred lower transcriptional activity than the shorter alleles in four different mammalian cell lines [30].

Both the 120 bp duplication [10,31] and the exon 3 VNTR [32-35] have also been associated with novelty seeking in a few studies. Although the association with novelty seeking is not as consistent as the association with ADHD and was not significant in a meta-analysis of available data [36], these reports remain of potential interest due the clinical association of ADHD with risk taking and stimulus seeking behaviours [37].

Here we set out to replicate these findings using a Taiwanese sample of 216 ADHD probands that had previously shown association to the dopamine transporter gene [38] (Brookes et al., in review). Grady and colleagues [19] suggest from their detailed analysis of sequence data across DRD4 that in Caucasian populations the 7-repeat allele is a relatively recent mutation that represents an independent clad and has been subject to positive selection. Their data suggests that in Asian populations the 2-repeat allele is a derivative of the Caucasian 7-repeat allele and we might therefore expect to see association between the 2-repeat allele and ADHD in Taiwanese populations [35,44]. This hypothesis was not supported by Qian et al, [17,18] who did observed a case-control association with *long *repeat alleles (4–6-repeat alleles; p < 0.05) in a Han Chinese population. This association, however, was not supported using within family tests of association and the association was not specific to any single allele. More recent data from Leung and colleagues does however support this hypothesis by observing a significant increase in prevalence of the 2-repeat allele in a Han Chinese ADHD sample in comparison to a control sample (p = 0.015) [45].

## Methods

216 ADHD probands between the ages of 5–15 years and available parents were recruited into the study from the Child Psychiatric Clinics in the Chang Gung Memorial Hospital in Taipei area, Taiwan. A total of 192 (83.6%) were males. IQ was 50–69 in 13%, 70–89 in 44%, 90–119 in 40% and >120 in 1%. The diagnosis of ADHD was made according to DSM-IV criteria following completion of a standard maternal interview (Kiddie-SADS) [39] and completion of parent and teacher Conner's revised rating scales [40]. In all 78% had the combined subtype and 22% the inattentive subtype of ADHD.

Genotyping was carried out using standard PCR methods and analysed on 2% agarose. Detection rate of DRD4 genotypes was 87.5% for the VNTR and 96% for the 5' 120 bp duplication. Both markers were in Hardy-Weinberg Equilibrium, and no Mendelian errors were observed. The family genotypes were analysed by single marker transmission disequilibrium test (TDT), and haplotype-based haplotype relative risk test (HHRR) run in UNPHASED . UNPHASED was also used to calculate haplotype associations for phase-certain haplotypes (TDTPHASE) and for uncertain haplotypes (HHRR) in addition linkage disequilibrium between the markers.

## Results

The TDT data revealed that neither marker investigated is associated with ADHD in this sample, either individually, or when combined together into a haplotype (Tables 1 and 2). The Global D' value between the two markers is low (0.2) suggesting that the two markers segregate independently from each other in this population. Refining the sample by removing those with IQ less than 70, did not improve the significance of the finding for either the exon III VNTR (TDT p = 0.55; HHRR p = 0.55), the 5'120 bp repeat (TDT p = 0.71; HHRR p = 0.73) or the haplotype of the two markers (TDT p = 0.45; HHRR p = 0.45). Subtype specific tests of association for the combined and inattentive subtypes analysed separately were non-significant (data not shown).

## Discussion

In summary we did not find the DRD4 markers to be associated with ADHD in a Taiwanese sample that has previously shown association with the dopamine transporter gene. As reported in previous studies in Asian populations the VNTR 7-repeat allele was absent and we also failed to replicate the previous reported associations in a Chinese population with 4-repeat and 6-repeat alleles [17,18] and in a Taiwanese population with the 2-repeat allele [45]. There have been no previously reported studies of the 120-bp repeat polymorphism and ADHD in Asian populations. Differences in association may also be due to differential diagnosis because of difference in cultures between western and eastern civilisations [48]. However the previously reported association with the dopamine transporter gene suggests that the clinical phenotype in this sample is comparable with samples ascertained in Europe, the United States and South America.

There are several possible explanations for the observed findings. First it is entirely possible that increased risk to ADHD associated with either of the markers studied may be absent in Asian populations. The association with the 7-repeat allele reported in Caucasian populations may be dependent on the presence of the 7-repeat allele itself and therefore absent from any population with low frequency of this allele. Critical sequences that increase risk for ADHD may therefore be absent in this population. The association with the 120-bp duplication has yet to be established and these data lend no further support to this potential finding. Second, our sample lacked power to detect very small main effects with less than 80% power at alpha = 0.05 for odds ratios less than 1.5 for associations with either the 2-repeat or 4-repeat alleles. Furthermore, it is feasible that environmental risk factors might interact with genetic risk factors reducing or abolishing main effects from genotype alone (e.g. [41,42]). In this case we would have little chance of detecting such associations unless we also had measured environmental risks. Third, previous studies using within family tests of association have failed to find evidence for association of the VNTR polymorphism with ADHD, whereas case-control designs have been more positive (discussed in [1]). The work of Holmes et al [8] using a collaborative dataset suggests that although there was no preferential transmission of the 7-repeat allele, there was a significant TDT association in the sub-group that had co-morbid conduct disorder. This is consistent with a report that parent-proband trios samples have a less severe clinical phenotype with lower levels of ADHD and co-morbid symptoms [43]. The association may therefore be present in co-morbid groups or groups which have certain aspects of the ADHD phenotype such as novelty seeking, cognitive impulsiveness [22], or persistence of symptoms [21] that have not been measured in this sample.

Fourth, it has been noted that for within family tests of association, dropped genotypes or genotyping errors for risk alleles with population frequency of less than 0.5 may give rise to false negative findings [49]. Although we were careful to rule out genotyping errors by identifying and re-genotyping ambiguous genotype calls, we did have a high level of dropping genotypes. Both proband and parental genotypes were however in Hardy-Weinberg Equilibrium suggesting that no major genotype biases were affecting this sample. We have attempted to deal with some limitations of within family tests of association by looking for case-control differences for the frequencies of the 2-repeat alleles. Using frequency in Han Chinese controls as 20% from data published by Leung and colleagues [45] and comparing this to the allele frequency of the 2-repeat of our probands (23.8%), there is no significant difference.

One final explanation may be due to hypothesized reciprocity between ADHD associations with the dopamine transporter and DRD4. As hypothesised by Swanson and colleagues [46] the risk allele for ADHD in the dopamine transporter may represent a hyper-efficient variant, whereas the risk alleles for the dopamine D4 receptor may represent a sub-sensitive variant, both leading to a hypo-dopaminergic system. Although additive or epistatic interactions between these two genes are likely, it might also be that they act independently of each other, describing two distinct causes of ADHD. It is therefore possible that samples that have been found to be associated with the DAT1 10-repeat risk allele, may not also be associated to the dopamine receptor D4 risk allele, due to over-representation of one allele or the other in particular populations. For example our UK sample that has also been found to be associated with DAT1 risk variants [47] (Brookes et al, in review), was not found to be associated with the VNTR polymorphism in the dopamine receptor D4 [15]. We investigated this possibility by re-analysing the DRD4 findings separately for individuals homozygous for the dopamine transporter risk allele and those with less than two risk alleles however there was no suggestion of association in these two sub-groups (data not shown).

## Conclusions

These results taken together with other reports of Taiwanese and Asian samples find no consistency in the association between genetic variants of DRD4 and ADHD. Further work in Taiwanese and other Asian populations will therefore be required to establish whether the reports of association of DRD4 genetic variants in Caucasian samples can be generalised to Asian populations.

## Abbreviations

**DRD4 **– Dopamine Receptor D4, **ADHD **– Attention Deficit Hyperactivity Disorder, **TDT **– Transmission Disequilibrium Test, **HHRR **– Haplotype-based Haplotype Relative Risk, **VNTR **– Variable Number Tandem Repeat.

## Competing interests

The author(s) declare that they have no competing interests.

## Authors' contributions

Keeley Brookes and Philip Asherson selected the markers for analysis. Keeley Brookes carried out the genotyping of the population and performed statistical analysis under the supervision of Philip Asherson. DNA was provided by Chih-Ken Chen, Yu-Shu Huang and Yu-Yu Wu. DNA was organised by Xiaohui Xu. Philip Asherson directed the study.

## Pre-publication history

The pre-publication history for this paper can be accessed here:



## Figures and Tables

**Table 1:** Allele Frequencies from HHRR analysis and Transmission ratio (T/NT) for TDT. Global and allele-specific tests of association were non-significant.

	*Case Frequency(%)*	*Control (NT) Frequency(%)*	*T/NT transmission*
5' marker			
120 bp	0.232	0.236	54 / 55
240 bp	0.768	0.764	179 / 178
			
Exon 3 VNTR			
2	0.234	0.244	46 / 48
3	0.005	0.000	1 / 0
4	0.751	0.736	149 / 146
5	0.010	0.020	2 / 4

**Table 2:** Haplotype analysis of the two markers did not find either global or haplotype-specific evidence for association.

*Haplotype*	*Case (T)*	*Frequency*	*Control (NT)*	*Frequency*	*OR*
1–2	17	0.095	12	0.068	1
1–4	21	0.119	26	0.15	0.57
2–2	25	0.144	31	0.176	0.59
2–3	1	0.006	0	0	0.72
2–4	110	0.625	103	0.583	0.77
2–5	2	0.011	4	0.36	0.36
